# Anti-SARS-CoV-2 antibodies following vaccination are associated with lymphocyte count and serum immunoglobulins in SLE

**DOI:** 10.1177/09612033231151603

**Published:** 2023-01-11

**Authors:** John A Reynolds, Sian E Faustini, Sofia Tosounidou, Tim Plant, Mandeep Ubhi, Rebecca Gilman, Alex G Richter, Caroline Gordon

**Affiliations:** 1 Rheumatology Research Group, Institute of Inflammation and Ageing, College of Medical and Dental Sciences, 1724 University of Birmingham, Birmingham, UK; 2 Rheumatology Department, 1731 Sandwell and West Birmingham NHS Trust, Birmingham UK; 3 Clinical Immunology Service, Institute for Immunology and Immunotherapy, 1724 University of Birmingham, Birmingham, UK

**Keywords:** Systemic lupus erythematosus, COVID-19, vaccine response, antibodies

## Abstract

**Objectives:**

Patients with Systemic Lupus Erythematosus are known to have dysregulated immune responses and may have reduced response to vaccination against COVID-19 while being at risk of severe COVID-19 disease. The aim of this study was to identify whether vaccine responses were attenuated in SLE and to assess disease- and treatment-specific associations.

**Methods:**

Patients with SLE were matched by age, sex and ethnic background to healthcare worker healthy controls (HC). Anti-SARS-CoV-2 spike glycoprotein antibodies were measured at 4–8 weeks following the second COVID-19 vaccine dose (either BNT162b2 or ChAdOx1 nCoV-19) using a CE-marked combined ELISA detecting IgG, IgA and IgM (IgGAM). Antibody levels were considered as a continuous variable and in tertiles and compared between SLE patients and HC and associations with medication, disease activity and serological parameters were determined.

**Results:**

Antibody levels were lower in 43 SLE patients compared to 40 HC (*p* < 0.001). There was no association between antibody levels and medication, lupus disease activity, vaccine type or prior COVID infection. Higher serum IgA, but not IgG or IgM, was associated with being in a higher anti-SARS-CoV-2 antibody level tertile (OR [95% CI] 1.820 [1.050, 3.156] *p* = 0.033). Similarly, higher lymphocyte count was also associated with being in a higher tertile of anti-SARS-CoV-2 (OR 3.330 [1.505, 7.366] *p* = 0.003)

**Conclusion:**

Patients with SLE have lower antibody levels following 2 doses of COVID-19 vaccines compared to HC. In SLE lower lymphocyte counts and serum IgA levels are associated with lower antibody levels post vaccination, potentially identifying a subgroup of patients who may therefore be at increased risk of infection.

## Introduction

Patients with rare autoimmune rheumatic diseases, including systemic lupus erythematosus (SLE), have an increased risk of infection with SARS-CoV-2 and an increased risk of death due to COVID-19.^1^ In a large study of over 2000 patients with SLE and COVID-19, lupus patients were more likely to require hospitalisation or mechanical ventilation, have concomitant sepsis or thromboembolic disease (including venous thromboembolism or stroke) than the general population.^2^

The rapid development and administration of vaccines against SARS-CoV-2 has been an important step in reducing the risk of severe COVID-19 in patients with SLE. In the general population, the BNT162b2 (Pfizer/BioNTech) and ChAdOx1 nCoV-19 vaccines reduced the risk of severe infection (requiring hospitalisation) in the early post-vaccination period by around 90% and 84%, respectively.^3,4^ The efficacy of such vaccines in patients with SLE is likely to depend at least in part on the seroconversion rate and the magnitude of the antibody response.

It is recognised that patients with SLE may have partial or incomplete serological responses to vaccines. Seroconversion rates to both influenza and pneumococcal vaccine in patients with SLE is variable and is dependent on the specific strains used.^5^ Whilst seroconversion, for example, to the influenza vaccine, seems to be globally reduced in SLE, other factors are likely to be important including disease activity^6^ and immunosuppressant medication.^7^

In the general population, there is an inverse association between anti-SARS-CoV-2 spike protein antibodies and symptomatic COVID-19.^8^ In 630 patients with systemic autoimmune rheumatic diseases, including 49 patients with SLE, non-responders (defined by low antibody titres) were more likely to develop COVID-19, independently of medication use.^9^ Measurement of anti-SARS-CoV-2 spike protein antibodies can therefore provide insight into the risk of developing COVID-19.

The aim of this study was to measure post-vaccination anti-SARS-CoV-2 antibody levels using an ELISA which detects combined IgG, IgA and IgM (IgGAM) antibodies, not IgG alone, in patients with SLE compared to healthy controls.

## Methods

### Study population

Patients with SLE who met either the 1997 Updated American College of Rheumatology (ACR) or 2012 Systemic Lupus International Collaborating Clinics (SLICC) Classification Criteria were recruited from Sandwell and West Birmingham NHS Trust (see supplementary methods for details) 4–8 weeks following their second SARS-CoV-2 vaccine dose. All study visits were conducted between 31st March and 31st August 2021. Disease activity was measured using the BILAG-2004 index^10^ and routine clinical and serological tests were conducted according to local protocols.

Fully anonymised healthy control data, from healthcare professionals, was obtained from the COVID-19 Convalescent Immunity (COCO) study^11^ and matched to the patient data by age, sex and ethnicity. All healthy controls donated blood samples 4 weeks after the 2nd vaccine dose.

### Anti-SARS-CoV2 IgGAM assay

Serum anti-SARS-CoV-2 trimeric spike (S) glycoprotein antibodies were measured using a validated ELISA that detects IgG, IgA and IgM (IgGAM) (anti-S-IgGAM) (product code: MK654; The Binding Site [TBS]) and presented as a ratio, as described previously.^12^ The sensitivity and specificity of this assay in COVID-19 infection is 94.7% and 98.4%, respectively. Positive antibody ratios were those ≥1.

### Ethical approval

This study was approved by Wales REC 6 (Ref 20/WA/0228). Approval for the COCO study was granted by London – Camden and Kings Cross REC (Ref 20/HRA/1817). All participants provided informed written consent and the research was conducted in compliance with the Declaration of Helsinki.

### Statistical analysis

Non-parametric descriptive statistics (Mann–Whitney U test and chi-squared test) were used for continuous and binary variables as appropriate. As the assumptions for linear regression models were not met, the anti-SARS-Cov2 antibody levels were converted into tertiles and univariate ordered logistic regression was conducted. Statistical analyses were performed using STATA/SE v.17.

## Results

We recruited 43 patients with SLE who were matched by age, sex and ethnicity to 40 healthy control (HC) healthcare workers from the COCO study, with the exception of older healthy volunteers from African/Caribbean backgrounds where there were insufficient numbers for 1:1 matching. The demographic features of the healthy volunteers and SLE patients are reported in Table 1.

**Table 1.** Demographics of the study population

	Healthy control (n = 40)	SLE (n = 43)	*p*
Age (years)	50.5 (44.5, 56.0)	51.0 (44.0, 60.0)	0.469
Gender (female)	35 (87.5%)	39 (90.7%)	0.640
Ethnicity			0.560
White	27 (67.5%)	24 (55.8%)	
African/Caribbean	4 (10%)	9 (20.9%)	
Asian	7 (17.5%)	8 (18.6%)	
Other	2 (5.0%)	2 (4.7%)	
Vaccine received			0.001
BNT162b2	40	33	
ChAdOx1 nCoV-19	0	10	
Time between 1st and 2nd dose (days)	77 (72, 84)	77 (71, 84)	0.913
Time between 2nd vaccine dose and sample collection (days)	32 (28, 35)	40 (31, 49)	<0.001
Disease duration (years) (n = 41)		18.5 (12.5, 24.4)	
Active disease (BILAG A or B score)		14 (36.6%)	
Prior COVID infection		9 (20.9%)	
Medication			
Anti-malarial		36 (83.7%)	
Oral prednisolone		29 (67.4%)	
Prednisolone dose		5 (5, 7.5)	
Immunosuppressant		22 (51.2%)	
Azathioprine		4 (9.3%)	
Mycophenolate mofetil		11 (25.6%)	
Methotrexate		4 (9.3%)	
Calcineurin inhibitor		4 (9.3%)	
Prior rituximab (ever)		8 (18.6%)	
Rituximab (up to 365 days before 2nd dose)		2 (5.0%)	
Number of cycles of rituximab		2(1, 3)	
Number of days between last RTX dose and 2nd vaccine dose		917 (355, 1373.5)	

BILAG; British Isles Lupus Assessment Group Index 2004 (see methods). For variables present in both HC and SLE patients, comparisons were made using Mann–Whitney U tests. *p* < 0.05 was considered statistically significant.

Most patients were receiving hydroxychloroquine (HCQ) (36, 87.3%) and oral prednisolone (29, 67.4%) with a median (IQR) dose of 5 (5, 7.5) mg/day. The most used immunosuppressant was mycophenolate mofetil (MMF) (11, 25.6%). A total of 8 patients had received rituximab (RTX) prior to the vaccine course; of these only 2 (25%) had RTX in the 365 days prior to the 2nd vaccine dose. One patient received rituximab between the 1st and 2nd vaccine dose.

### Anti-SARS-CoV-2 IgGAM in patients with SLE

**Figure 1.**
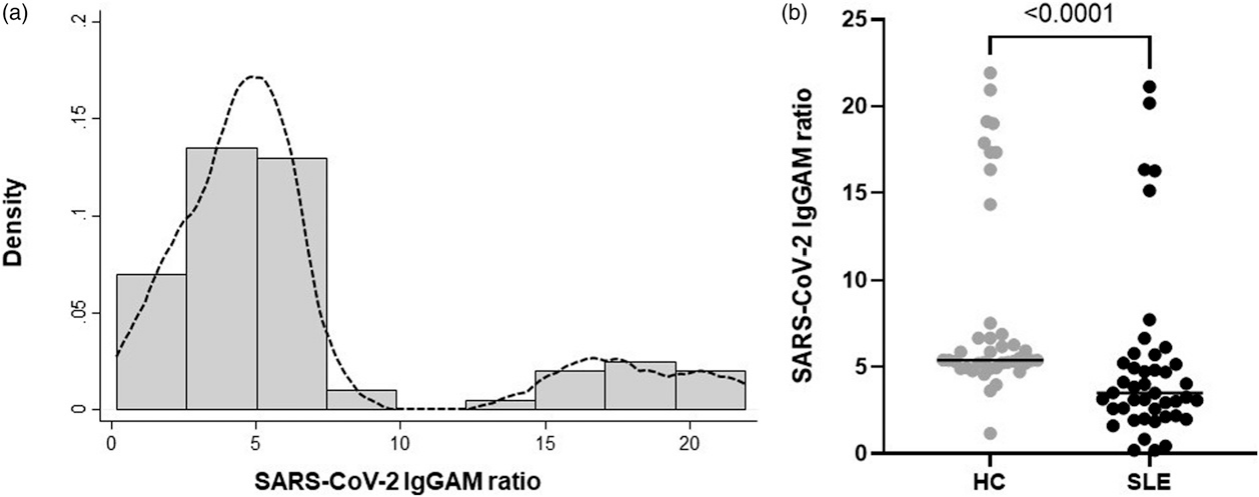
Anti-SARS-CoV-2 IgGAM following vaccination is lower in patients with SLE. (a) Bimodal distribution of anti-SARS-CoV-2 antibodies with a smaller number of participants with high levels. Line on histogram shows kernel density estimation. (b) Lower ratios of anti-SARS-CoV-2 antibody in patients with SLE compared to HC. Horizontal bars show median ratios. Comparison using Mann–Whitney U test.

Positive antibody ratios (≥1) were observed in all HC compared to 40/43 (93.0%) patients with SLE (chi^2^, *p* = 0.089). The distribution of the antibody level appeared to be bimodal, although the number of participants with higher antibody ratios was modest, representing 14/83 (16.9%) of the cohort (Figure 1 and see supplementary data). In SLE patients, the median (IQR) time between 2nd vaccine dose and antibody testing was 40 (31, 49) days compared to 32 (28, 35) days for HC (Mann–Whitney U test *p* < 0.001). In SLE patients, there was no correlation between the time from vaccine to blood draw, and anti-SARS-CoV-2 antibody levels (Spearman r = −0.118, *p* = 0.453).

Overall, anti-SARS-CoV-2 antibody ratios were lower in SLE (3.51 [2.58, 5.71]) compared to HC (5.39 [5.08, 7.20]), (*p* < 0.001) (Figure 1). There was no significant difference in antibody ratios in participants who reported prior COVID-19 infection (4.73 [3.10, 6.65]) compared to those that did not (3.20 [2.13, 4.94]) (*p* = 0.732) (see supplementary data).

There was no difference in antibody ratios in SLE patients between ethnic groups: White (3.10 [2.19, 4.73]), African/Caribbean (3.99 [3.51, 6.28]), Asian (5.47 [3.32, 6.21]), and Other (1.97 [1.93, 2.01]), *p* = 0.116. Similarly, there was no difference between antibody ratios in patients who received two doses of the BNT162b2 (Pfizer/BioNTech) (3.51 [2.19, 5.22]) vaccine compared to those patients who received two doses of ChAdOx1 nCoV-19 (3.12 [2.60, 4.82]) (*p* = 0.810).

There were also no differences between patients receiving MMF or any other immunosuppressant drug, oral prednisolone or HCQ (Supplementary Table S1). Only 8 (18.6%) patients had received RTX prior to the 1st vaccine dose with a median time of 917 (355, 1373.5) days from RTX last dose to sample collection. There was no difference in the antibody ratios in patients who had received RTX (2.10 [0.645, 4.78]) compared to those who had not (3.51 [2.62, 5.22]) (*p* = 0.109), nor any correlation between the antibody ratios and time of last dose.

### Anti-SARS-CoV-2 antibodies in SLE were associated with serum IgA and lymphocyte count

In univariate ordered logistic regression models, levels of serum IgA were associated with being in a higher anti-SARS-CoV-2 antibody tertile (OR [95% CI] 1.820 [1.050, 3.156] *p* = 0.033) (Table 2). There was no significant association with IgG (OR 1.132 [0.983, 1.303] *p* = 0.084) or IgM (OR 1.975 [0.743, 5.314] *p* = 0.178). Patients who had received RTX had lower levels of IgA (1.59 [1.31, 2.21] vs 2.46 [1.73, 3.43], *p* = 0.035) and IgM (0.37 [0.25, 0.62] vs 0.87 [0.56, 1.41], *p* = 0.006] compared to those that had not. When rituximab use was included in the ordered logistic regression mode, the association between serum IgA and anti-SARS-CoV-2 tertile was no longer statistically significant (OR 1.68 [0.947, 2.995], *p* = 0.076).

**Table 2.** Univariate ordered logistic regression model of anti-SARS-CoV-2 antibody levels in patients with SLE

	Odds ratio	95% CI	*p*
Demographic variables			
Age (years)	1.002	0.952, 1054	0.948
Male	0.922	0.146, 5.813	0.931
Disease duration (years)	1.014	0.950, 1.082	0.683
Pfizer/BioNTech BNT162b2 vaccine	1.098	0.272, 4.429	0.895
Prior COVID infection	2.131	0.525, 8.661	0.290
Active disease (BILAG A or B score)	1.261	0.359, 4.430	0.718
Medication			
Anti-malarial	4.677	0.508, 43.057	0.173
Prednisolone	0.695	0.205, 2.354	0.559
Any immunosuppressant	1.326	0.410, 4.289	0.637
Mycophenolate mofetil	1.521	0.425, 5.442	0.519
Immunosuppressant + prednisolone	1.349	0.417, 4.358	0.617
Rituximab (prior to vaccination)	0.332	0.061, 1.807	0.202
Serum immunoglobulins			
IgG (g/l)	1.132	0.983, 1.303	0.084
**IgA (g/l)**	1.820	1.050, 3.156	0.033
IgM (g/l)	1.975	0.743, 5.314	0.178
Haematological parameters			
Haemoglobin (g/dl)	0.654	0.402, 1.066	0.088
Total white cell count (10^9^ cells/l)	1.201	0.869, 1.661	0.267
**Lymphocytes (10**^**9**^** cells/l)**	**3.330**	**1.505, 7.366**	**0.003**
Neutrophils (10^9^ cells/l)	0.860	0.622, 1.190	0.363
Platelets (10^9^ cells/l)	1.005	0.995, 1.016	0.323

Anti-SARS-CoV-2 antibody levels were split into tertiles and univariate ordered logistic regression models were used with antibody tertile as the dependent variable such that the values in the table are the odds of being in a higher antibody tertile for each unit increase in the independent variable. *p* < 0.05 was considered to be statistically significant

There was also a significant association between blood lymphocyte count and anti-SARS-CoV-2 tertile (OR 3.330 [1.505, 7.366] *p* = 0.003) which was not observed for other haematological parameters (Table 2). This observation remained statistically significant after adjustment in individual models for important confounders which might influence lymphocyte count: ethnicity (OR 3.400 [1.314, 8.800] *p* = 0.012), previous rituximab use (OR 3.937 [1.646, 9.424], *p* = 0.002) or use of other immunosuppressants (OR 3.371 [1.520, 7.478], *p* = 0.003).

## Discussion

This is the first study to measure combined anti-SARS-CoV2 antibody responses following COVID-19 vaccination in patients with SLE with an assay that detects combined IgG, IgA and IgM antibodies. In our study, the distribution of anti-SARS-CoV-2 appeared bimodal with around 17% of the participants having notably higher antibody ratios. Compared to HC, patients with SLE had lower median ratios and wider variability (IQR) of antibodies. A bimodal pattern has been observed by others in patients with solid malignancy, and in patients who have received haematopoietic stem cell transplants, although in these patient groups, the distributions were low/normal rather than normal/high.^13^ Whilst it is recognised that patients with SARS-CoV-2 infection prior to vaccination may have higher antibody ratios, we did not observe this association in our study. In a large general population study by Ali et al.,^14^ anti-SARS-CoV-2 IgG and IgA were significantly higher in people with confirmed prior infection than those without, although some participants had notably higher antibody levels without any prior history of infection. Although we used self-reported prior COVID infection rather than PCR confirmation (which was not routinely available at the time), our study supports the observation that some individuals may have high post-vaccine antibody levels without prior infection. It is likely that factors other than prior infection resulted in a small number of participants with high anti-IgGAM levels. A recent study by Mentzer et al.^15^ identified that individuals with the HLA-DQB1*06 genotype had significantly higher post vaccine antibody levels. As our assay measured IgGAM, it is possible that the timing of sample collection following the second vaccine dose is important as, for example, IgM antibodies would be expected to wane over this time period. However, we did not find any association between antibody levels and the time between 2nd vaccine dose and sample collection.

Lower post-vaccine antibody levels in patients with SLE have been reported in other studies. A large study by Furer et al.^16^ of 686 autoimmune inflammatory rheumatic disease patients (AIIRD) (including 101 with SLE) and 121 general population controls demonstrated lower seropositivity rates and anti-SARS-CoV-2 IgG antibody titres, 2–6 weeks after the second BNT162b2 (Pfizer/BioNTech) vaccine dose, in SLE patients compared to controls. Across the AIIRD cohort, the seropositivity rate was lower in patients receiving MMF, methotrexate, glucocorticoids or abatacept. In 126 SLE patients in which samples were obtained 15 days after the second dose, both MMF and MTX use were associated with lower SARS-CoV-2 antibody titres.^17^ In our study, we did not observe a relationship between medication and antibody titre although our study is likely to be statistically underpowered to detect these differences as there were small numbers of patients taking each individual medication.

In contrast to the study by Moyon et al. (which excluded patients previously treated with RTX), we observed an association between the anti-SARS-CoV-2 IgGAM titre and serum IgA and lymphocyte count. Moyon et al.,^17^ reported that antibody responses were associated with total IgG but not IgA or IgM levels. Whilst the study above measured anti-SARS-CoV-2 IgG, our assay measured IgG, IgA and IgM which may explain the observed association with IgA levels. Although this observation was not statistically significant after adjustment for prior rituximab use, it should be noted that serum IgA levels do not typically reduce significantly following rituximab.^18^

Lymphopenia is common in patients with SLE due to active disease and/or effects of immunosuppressive therapy. We identified a positive independent association between antibody levels and total lymphocyte count, but not other haematological parameters. Our findings support observations in patients with haematological malignancy^19^ and multiple sclerosis.^20^ Flow cytometry studies following the 2nd vaccine dose in patients with SLE identified that the total number of B cells and proportion of naïve B cells pre-vaccine were associated with anti-SARS-CoV-2 IgG.^17^ In our study, standard routine clinical measurement of lymphocytes, rather than detailed flow cytometry, was sufficient to demonstrate an association with antibody levels. This is particularly interesting as only around 10% of circulating lymphocytes are typically B cells. This observation should be confirmed in an independent data set.

There are some important limitations of this study. Firstly, this is a small study and thus would not have the statistical power to identify associations between medication and anti-SARS-CoV2 antibody levels. Both IgA and lymphocyte count were measured post-vaccination (at the time of antibody measurement) and further studies are needed to determine that pre-vaccine levels can predict antibody responses. Finally, in this study, we were not able to report the clinical outcomes of any subsequent COVID-19 infection in either the HC or SLE.

In conclusion, patients with SLE have reduced antibody responses to two doses of COVID-19 vaccine (either BNT162b2 or ChAdOx1 nCoV-19) compared to healthy controls. Lower post-vaccine serum IgA levels or lymphocytes counts are associated with reduced antibody responses and may indicate a subgroup of patients that have a more significant immunodeficiency and have a reduced response to vaccination. This may allow more tailored advice on risk, infection avoidance strategies and access to therapeutics.

## Supplemental Material

Supplemental Material - Anti-SARS-CoV-2 antibodies following vaccination are associated with lymphocyte count and serum immunoglobulins in SLE Click here for additional data file. Supplemental Material for Anti-SARS-CoV-2 antibodies following vaccination are associated with lymphocyte count and serum immunoglobulins in SLE by John A Reynolds, Sian E Faustini, Sofia Tosounidou, Tim Plant, Mandeep Ubhi, Rebecca Gilman, Alex G Richter and Caroline Gordon in Lupus
