# Supplementary material for: Anti-SARS-CoV-2 antibodies following vaccination are associated with lymphocyte count and serum immunoglobulins in SLE
Source: Lupus. 2023 Jan 11;32(3):431–7. doi: 10.1177/09612033231151603 (PMC9843147; doi:10.1177/09612033231151603)
Supplement: Supplemental Material - Anti-SARS-CoV-2 antibodies following vaccination are associated with lymphocyte count and serum immunoglobulins in SLE [file sj-pdf-1-lup-10.1177_09612033231151603.pdf]

Supplementary methods

Patients were recruited from clinics at Sandwell and West Birmingham NHS Trust. All patients were required to fulfil either the 1997 Updated American College of Rheumatology (ACR) Classification Criteria [1] or the 2012 Systemic Lupus International Collaborating Clinics (SLICC) Classification Criteria [2].

1. Hochberg MC. Updating the American College of Rheumatology revised criteria for the classification of systemic lupus erythematosus. Arthritis and rheumatism 1997;40(9):1725.

2. Petri M, Orbai A-M, Alarcón GS, et al. Derivation and validation of the Systemic Lupus International Collaborating Clinics classification criteria for systemic lupus erythematosus. Arthritis and rheumatism 2012;64(8):2677-86.

Blood samples for routine clinical parameters, serology and for anti-SARS-CoV-2 antibodies were collected at the time of disease activity assessment.

Supplementary data

Supplementary table S1: Levels of anti-SARS-CoV-2 antibody and immunosuppressant medications in patients with SLE

	Yes	No	p	Correlation, r	p
Medication					
Anti-malarial (n=36)	3.78 (2.39, 5.47)	3.08 (1.98, 3.85)	0.468		
Prednisolone (n=29)	3.25 (2.01, 5.15)	3.77 (2.95, 5.22)	0.513		
Any immunosuppressant (n=22)	3.38 (2.60, 5.71)	3.50 (2.13 4.73)	0.692		
Mycophenolate mofetil (n=11)	4.70 (0.45, 5.15)	3.32 (2.39, 5.47)	>0.999		
Immunosuppressant + prednisolone (n=19)	3.51 (2.19, 5.71)	3.32 (2.36, 4.98)	0.818		
Rituximab (prior to vaccination) (n=8)	2.10 (0.645, 4.78)	3.51 (2.62, 5.22)	0.109		
Time from rituximab to sample collection (days) (n=9)*				-0.524	0.183

Comparisons made with Mann-Whitney U tests or Spearman's r.

*1 patient received rituximab between 1st and 2nd COVID vaccine dose

Supplementary Figure S1: Distribution of anti-SARS-CoV-2 IgGAM in healthy controls and patients with SLE

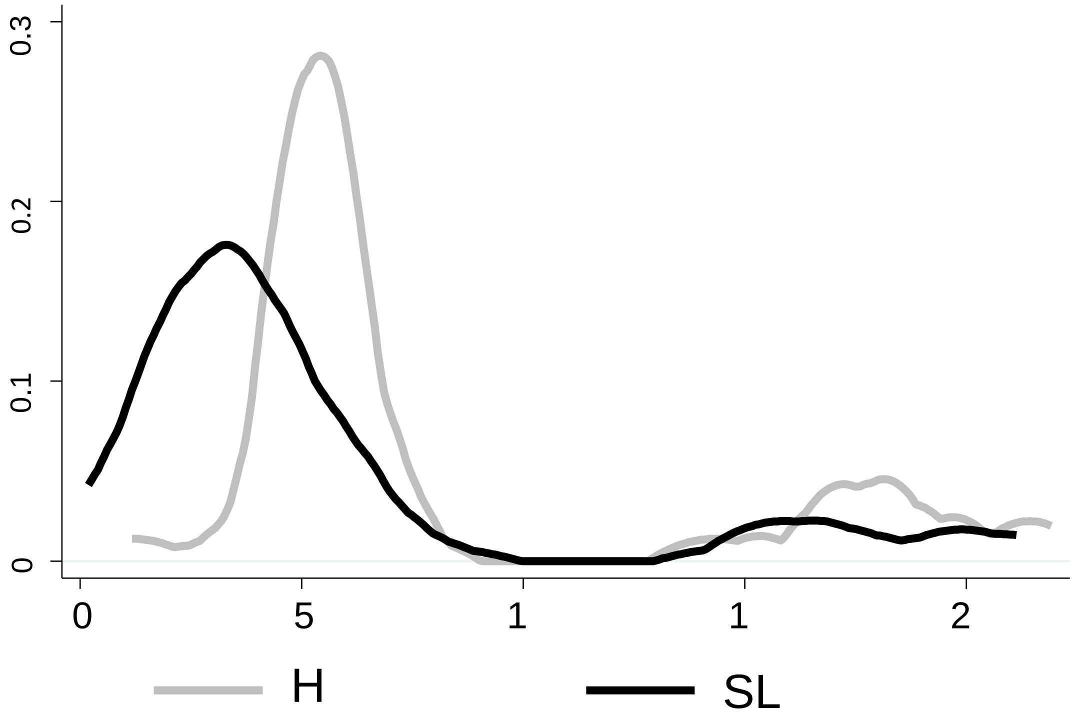

Supplementary Figure S2: Levels of anti-SARS-CoV-2 IgGAM following the second dose in healthy controls and patients with SLE according to previous COVID-19 infection

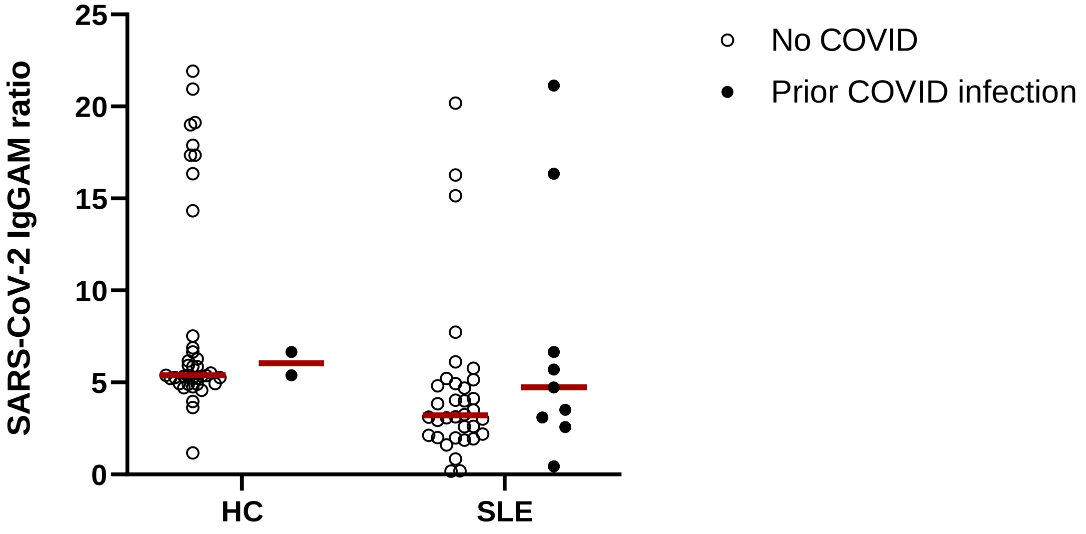

Levels of anti-SARS-CoV-2 IgGAM in healthy controls (HC) and patients with SLE according to history of prior COVID infection. The horizontal bar shows the median for the group.
